# Secreted APE1/Ref-1 inhibits TNF-α-stimulated endothelial inflammation via thiol-disulfide exchange in TNF receptor

**DOI:** 10.1038/srep23015

**Published:** 2016-03-11

**Authors:** Myoung Soo Park, Sunga Choi, Yu Ran Lee, Hee Kyoung Joo, Gun Kang, Cuk-Seong Kim, Soo Jin Kim, Sang Do Lee, Byeong Hwa Jeon

**Affiliations:** 1 Infectious Signaling Network Research Center and Research Institute for Medical Sciences, Department of Physiology, School of Medicine, Chungnam National University, Daejeon, 301-747, Republic of KOREA

## Abstract

Apurinic apyrimidinic endonuclease 1/Redox factor-1 (APE1/Ref-1) is a multifunctional protein with redox activity and is proved to be secreted from stimulated cells. The aim of this study was to evaluate the functions of extracellular APE1/Ref-1 with respect to leading anti-inflammatory signaling in TNF-α-stimulated endothelial cells in response to acetylation. Treatment of TNF-α-stimulated endothelial cells with an inhibitor of deacetylase that causes intracellular acetylation, considerably suppressed vascular cell adhesion molecule-1 (VCAM-1). During TSA-mediated acetylation in culture, a time-dependent increase in secreted APE1/Ref-1 was confirmed. The acetyl moiety of acetylated-APE1/Ref-1 was rapidly removed based on the removal kinetics. Additionally, recombinant human (rh) APE1/Ref-1 with reducing activity induced a conformational change in rh TNF-α receptor 1 (TNFR1) by thiol-disulfide exchange. Following treatment with the neutralizing anti-APE1/Ref-1 antibody, inflammatory signals via the binding of TNF-α to TNFR1 were remarkably recovered, leading to up-regulation of reactive oxygen species generation and VCAM-1, in accordance with the activation of p66^shc^ and p38 MAPK. These results strongly indicate that anti-inflammatory effects in TNF-α-stimulated endothelial cells by acetylation are tightly linked to secreted APE1/Ref-1, which inhibits TNF-α binding to TNFR1 by reductive conformational change, with suggestion as an endogenous inhibitor of vascular inflammation.

Chronic vascular inflammation plays a key role in the pathogenesis of atherosclerosis and other vascular disease^1^. Accordingly, the regulation of inflammatory reactions in the vascular endothelium is a potential target for therapeutic intervention in the treatment of chronic inflammation, such as atherosclerotic disease. Inflammation is mainly mediated by monocyte adhesion to endothelial cells. The recruitment of monocytes to the affected tissue and accumulation of monocyte-derived phagocytes^2^ are actively mediated and precisely controlled by cytokines, such as interleukin-1β (IL-1β), IL-6, IL-8, and tumor necrosis factor (TNF)-α. The interaction between blood monocytes and the vascular endothelium involves a cytokine-mediated process that includes monocyte rolling, arrest, firm adhesion, and diapedesis^3^. During vascular inflammation, the adhesion cascade of monocytes is regulated by a combination of endothelial cell surface adhesion molecules including vascular cell adhesion molecule-1 (VCAM-1), intercellular cell adhesion molecule-1, and E-selectin^4^.

In vascular inflammatory responses, TNF-α which is released from macrophages exert direct effects on a multitude of secondary inflammatory mediators via binding with the TNF-α receptors (mainly TNFR1)^5^, resulting in the production of reactive oxygen species (ROS) and the activation of nuclear factor-κB (NF-κB)^6^,^7^. Activated NF-κB in the nucleus regulates the transcription of genes involved in the pathogenesis of inflammatory lesions, including cytokines, chemokines and adhesion molecules^8^. Therefore, the treatment of vascular inflammation with agents that block initial TNF-α activity can be highly beneficial and can minimize side effects or the disruption of overlapping intracellular signaling. For example, three representative drugs, infliximab, adalimumab, and etanercept, are TNF-α antibodies or TNFR1-Fc chimeras and function to prevent TNF-α from binding to its receptor; all are currently used to treat inflammatory disease^9^. Although TNF inhibition fails to improve symptoms in severe late-stage infectious diseases, trials are necessary to evaluate its use in vascular inflammatory diseases. TNFR1 is a member of the TNF receptor superfamily, which is a group of cytokine receptors that have the ability to bind TNFs via an extracellular cysteine-rich domain (CRD)^10^. TNFR1 has six consensus cysteine residues forming three disulfide bonds in each of the four CRDs for recognition of its ligand, homotrimeric TNF-α^11^,^12^. Considering the structure of the TNF-α/TNFR1 complex, some studies have reported the development of TNF-α inhibitors based on the key sites of the TNF-α/TNFR1 interaction, peptide mimics of the TNFR1 loop, or small molecules that bind to TNF-α directly^13^.

Apurinic apyrimidinic endonuclease 1/Redox factor-1 (APE1/Ref-1, also known as Ref-1) is a multifunctional protein; its N-terminal region is involved in redox activity and regulates multiple transcription factors, and its C-terminus is involved in base excision DNA repair activity^14^. APE1/Ref-1 undergoes active shuttling between the cytoplasm and nucleus in response to oxidative stress^15^,^16^,^17^. Interestingly, previous studies, including ours, have reported the possibility for the extracellular secretion of APE1/Ref-1. Auto-antibodies against APE1/Ref-1 have been found in patients with systemic lupus erythematosus^18^ and lung cancer^19^, suggesting the exposure of APE1/Ref-1 to the host immune system. Elevated levels of APE1/Ref-1 were also observed in the blood of endotoxemic rats^20^ and in bladder cancer^21^, implying that APE1/Ref-1 functions as a secreted protein.

Because the level of secreted APE1/Ref-1 is substantially increased in response to acetylation, we hypothesized that secreted APE1/Ref-1 could be an effective regulator in inflammatory reactions via its reduction. We tested this hypothesis using TNF-α-treated human umbilical vein endothelial cells (HUVECs) as a vascular inflammation model. We provide compelling experimental evidence to indicate that extracellular secreted APE1/Ref-1 in response to intracellular acetylation inhibits inflammatory signaling via a reduction in TNFR1, showing that treatment of anti-APE1/Ref-1 antibody in histone deacetylase inhibitor (HDACi), trichostatin A (TSA)-mediated modulation against TNF-α-stimulated endothelial activation recovers not only upregulation of adhesion molecule but also the generation of ROS.

## Results

### TSA treatment caused downregulation of VCAM-1 in TNF-α-stimulated HUVECs

**Figure 1:**
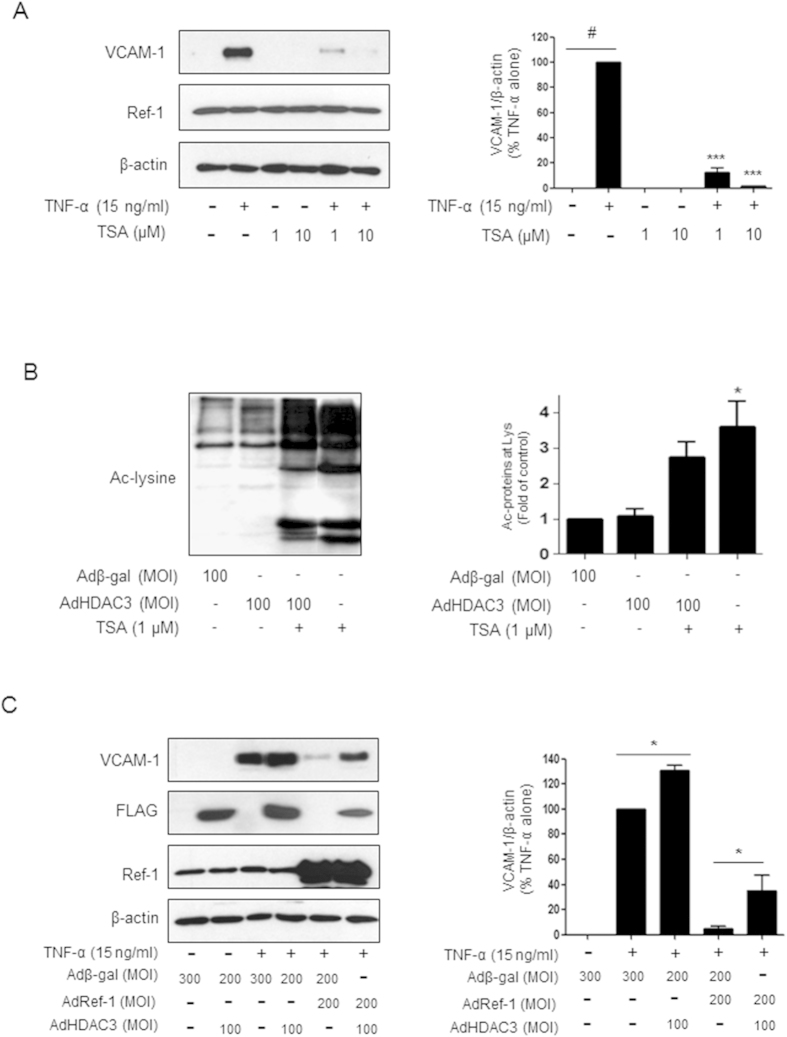
Trichostatin A (TSA) treatment caused downregulation of VCAM-1 expression in TNF-α-stimulated HUVECs. (**A**) Effect of trichostatin A (TSA)-mediated acetylation on VCAM-1 expression in TNF-α-stimulated human umbilical vein endothelial cells (HUVECs). Cells were treated with TSA (1, or 10 μM) in the presence or absence of TNF-α (15 ng/ml). (**B**) Acetylation and deacetylation profiles of intracellular proteins in response to 1 μM TSA treatment and histone deacetylase3 (HDAC3) expression, respectively. (**C**) Effects of APE1/Ref-1 or HDAC3 overexpression on VCAM-1 expression in TNF-α-stimulated HUVECs. Recombinant β-galactosidase adenovirus was used as a control. The blots were stripped and reprobed with anti-APE1/Ref-1 or β-actin antibodies to ensure protein loading. Representative blots are shown. Bar graph shows densitometry quantification of western blot data. The data are represented as % densitometry values of TNF-α-induced VCAM-1 expression. *Columns*, mean (n = 3); *bars*, SE. ***P < 0.001, significantly different from TNF-α-treated control cells; *P < 0.01 significantly different from cells expressing HDAC3; ^#^P < 0.01 significantly different from untreated control cells based on a one-way analysis of variance (ANOVA) followed by Dunnett’s tests. Similar results were observed in replicate experiments.

The HDACi, TSA inhibits the expression of the cell adhesion molecule VCAM-1 in TNF-α-stimulated endothelial cells^22^, but the sequence of events leading to anti-inflammatory effects in the vascular system is still unclear. Accordingly, we examined the mechanism of VCAM-1 suppression in TNF-α-stimulated endothelial cells treated with TSA. As shown in Fig. 1A,B, TSA treatment resulted in a considerable decrease in VCAM-1 expression and an increase in intracellular acetylation. The level of VCAM-1 was almost completely downregulated unlike cells simulated with TNF-α only (Fig. 1A).

To test whether TSA-mediated acetylation causes downregulation of VCAM-1 in TNF-α-stimulated endothelial cells, we observed the effect of deacetylase on VCAM-1 levels using an adenovirus expressing HDAC3. The level of VCAM-1 in TNF-α-treated cells was substantially increased in HDAC3-overexpressed cells presenting no acetylation (Fig. 1B,C). As shown in the bar graph in Fig. 1C, the constitutive expression of VCAM-1 increased to ~130% following HDAC3 adenoviral infection compared with that of TNF-α-stimulated cells. In contrast, the upregulated VCAM-1 caused by TNF-α treatment or additional HDAC3 adenoviral infection was considerably decreased by the introduction of adenoviral APE1/Ref-1 (Fig. 1C). The VCAM-1 level decreased by ~38% in response to the expression of APE1/Ref-1 based on densitometric scanning of the immunoreactive bands after correcting for the actin loading control. Collectively, these results indicated that VCAM-1 expression in TNF-α stimulated endothelial cells is regulated by acetylation/deacetylation, implying a functional role of the APE1/Ref-1 protein.

### Acetylated APE1/Ref-1 was secreted from TSA-treated endothelial cells and rapidly removed its acetyl moiety

**Figure 2:**
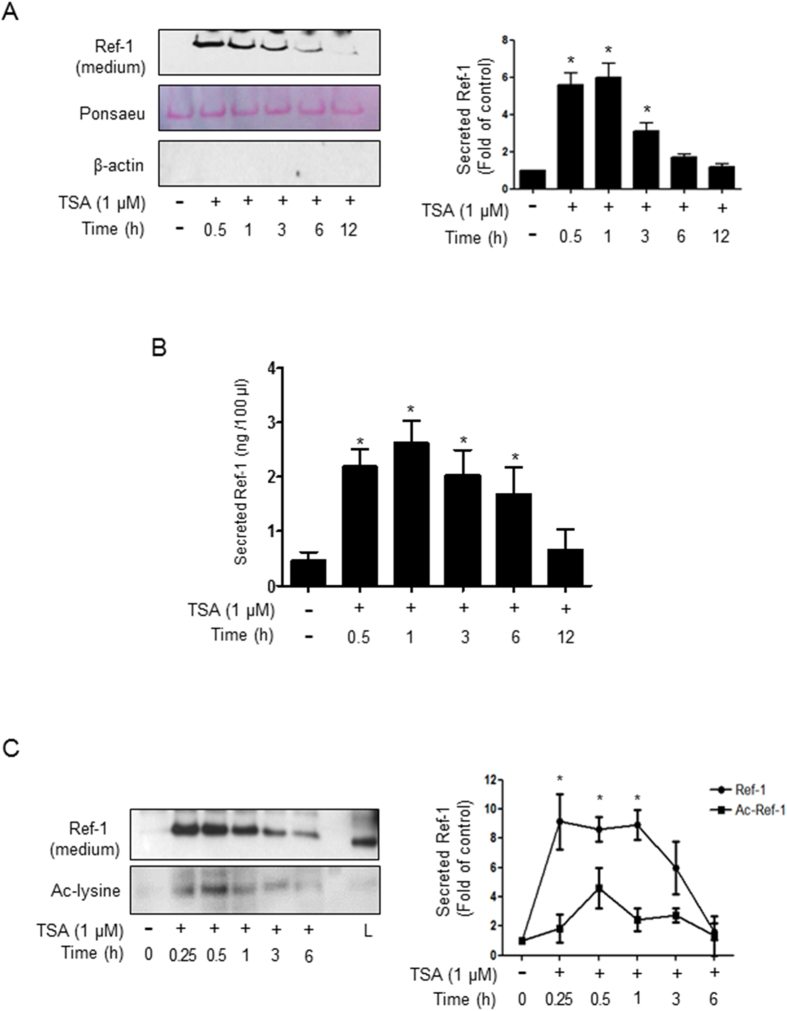
TSA treatment caused extracellular secretion of acetylated APE1/Ref-1 from TNF-α-stimulated HUVECs followed by rapid removal of acetyl moiety. (**A**) After cells were treated with TSA (1 μM) for the indicated times, secreted APE1/Ref-1 was precipitated chemically and confirmed by immunoblotting using anti-APE1/Ref-1 antibody. Unknown protein was demonstrated by Ponceau S staining to ensure equal protein loading and anti-β-actin antibody was used to demonstrate a lack of contamination of cellular proteins. (**B**) Quantitative analysis of secreted APE1/Ref-1 was performed by enzyme-linked immunosorbent assay. The amounts of secreted APE1/Ref-1 from TSA-treated cells are shown for each time point. (**C**) Removal kinetics of acetyl moiety after secretion of acetylated APE1/Ref-1. TNF-α-stimulated human umbilical vein endothelial cells (HUVECs) were treated with 1 μM TSA in a time-dependent manner. The conditioned medium was carefully collected without cell debris and immunoprecipitated with anti-APE1/Ref-1 followed by anti-acetyl-lysine or monoclonal anti-APE1/Ref-1 antibodies. L; cell lysate was used as a positive control. *Columns*, mean (n = 3); *bars*, SE. *P < 0.01, significantly different from TSA non-treated cells by a one-way ANOVA followed by Dunnett’s tests. These experiments were performed with similar results. Representative blots are shown.

In previous studies, we reported that acetylated APE1/Ref-1 (Ac-APE1/Ref-1) is secreted after intracellular acetylation in TSA-treated human embryonic kidney epithelial cells (HEK 293 T)^23^. We examined whether TSA-mediated acetylation also affects the secretion of APE1/Ref-1 in HUVECs. APE1/Ref-1 in the culture supernatant of HUVECs was chemically or immunologically analyzed. As shown in Fig. 2A, secreted APE1/Ref-1 was clearly detected in the supernatant of TSA-treated cells. Exposure to TSA resulted in a time-dependent and considerable increase in APE1/Ref-1. The levels of APE1/Ref-1 reached a plateau at 0.5–1 h after TSA treatment and then gradually declined. The extracellular level of secreted APE1/Ref-1 was quantitatively measured by ELISA. As shown in Fig. 2B, the level of secreted APE1/Ref-1 in the culture increased rapidly in response to TSA-mediated acetylation. The maximal value was 2.7 ng/100 μl of culture media at 1 h, and it gradually declined thereafter. These data indicated that TSA-mediated acetylation considerably induced the extracellular secretion of APE1/Ref-1.

To confirm whether APE1/Ref-1 secretion is regulated by acetylation, we examined the acetylation of secreted APE1/Ref-1 using anti acetyl-lysine antibody. TSA-mediated acetylation caused an initial increase in secreted Ac-APE1/Ref-1 compared with untreated control cells (Fig. 2C). Interestingly, the acetyl group was rapidly removed by 0.5 h, but the APE1/Ref-1 protein was still detected until 6 h, indicating the existence of the nonacetylated, native form of APE1/Ref-1. Neither APE1/Ref-1 nor its acetylated form was detected in TSA treated endothelial cells expressing HDAC3 (data not shown).

### Acetylation-mediated VCAM-1 suppression was recovered by the removal of APE1/Ref-1 in the supernatant of TNF-α-stimulated endothelial cells

**Figure 3:**
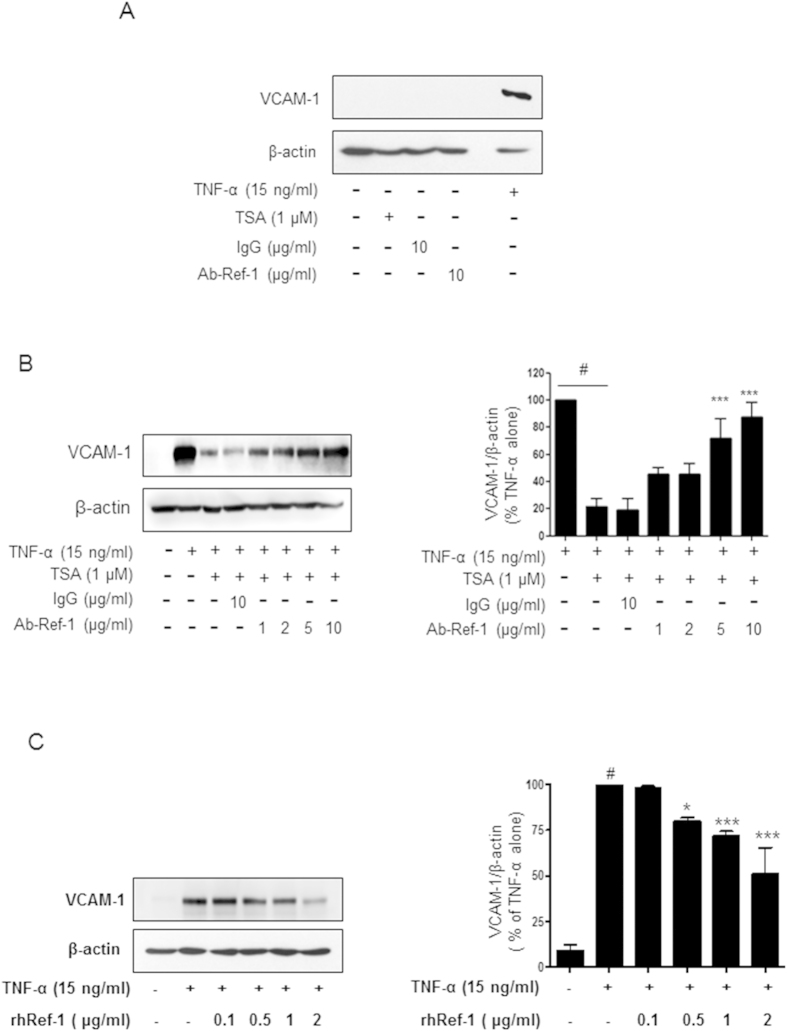
Neutralizing anti-APE1/Ref-1 antibody recovered TSA-mediated suppression of VCAM-1 expression in TNF-α-stimulated HUVECs. (**A**) Treatment with TSA, IgG, or anti-APE1/Ref-1 did not affect VCAM-1 expression in TNF-α-stimulated human umbilical vein endothelial cells (HUVECs) Cell lysates were obtained from TNF-α-stimulated HUVECs treated with TSA (1 μM), IgG (10 μg/ml), or anti-APE1/Ref-1(10 μg/ml). (**B**,**C**) Anti-APE1/Ref-1 antibody was dose-dependently pretreated and then 1 μM TSA was added (**B**). rh APE1/Ref-1 was pretreated for 0.5 h at the indicated concentrations (**C**) before stimulation of HUVECs with TNF-α. HUVEC lysates were obtained and immunoblotting for VCAM-1 was performed. The blots were stripped and reprobed with anti-β -actin antibody to ensure equal protein loading. Immunoblotting for each protein was performed three times using independently prepared lysates and similar results were obtained. *Columns*, mean (n = 3); *bars*, SE. *P < 0.05, ***P < 0.001, significantly different from TSA and TNF-α-treated cells; ^#^P < 0.01 significantly different from only TNF-α-treated cells by one-way ANOVA followed by Dunnett’s tests. Data shown are representative of replicate experiments with similar results.

To further examine the functional role of extracellular secreted APE1/Ref-1, we determined the effect of the anti-APE1/Ref-1 antibody on VCAM-1 expression in TNF-α-stimulated HUVECs. VCAM-1 was not detected after treatment with only TSA, IgG or anti-APE1/Ref-1, but was markedly increased in TNF-α-stimulated cells (Fig. 3A). As shown in Fig. 3B, TSA-mediated acetylation substantially suppressed TNF-α-induced VCAM-1 expression, and this suppression was markedly abrogated in a concentration-dependent manner by the removal of APE1/Ref-1 with neutralizing anti-APE1/Ref-1 antibody. These results clearly indicated that the regulation of VCAM-1 in response to acetylation/deacetylation is associated with the functional role of extracellular secreted APE1/Ref-1, rather than a direct effect of TSA. To evaluate the function of extracellular APE1/Ref-1, the effect of rh APE1/Ref-1 on TNF-α-induced VCAM-1 expression was determined. As shown in Fig. 3C, pretreatment with rh APE1/Ref-1 (0.5–2 μg/ml) suppressed TNF-α-induced VCAM-1 expression in a concentration-dependent manner, suggesting that extracellular secreted APE1/Ref-1 has an anti-inflammatory function.

### Neutralization of secreted APE1/Ref-1 recovered ROS generation, which was temporarily inhibited by TSA treatment in TNF-α-stimulated endothelial cells

**Figure 4:**
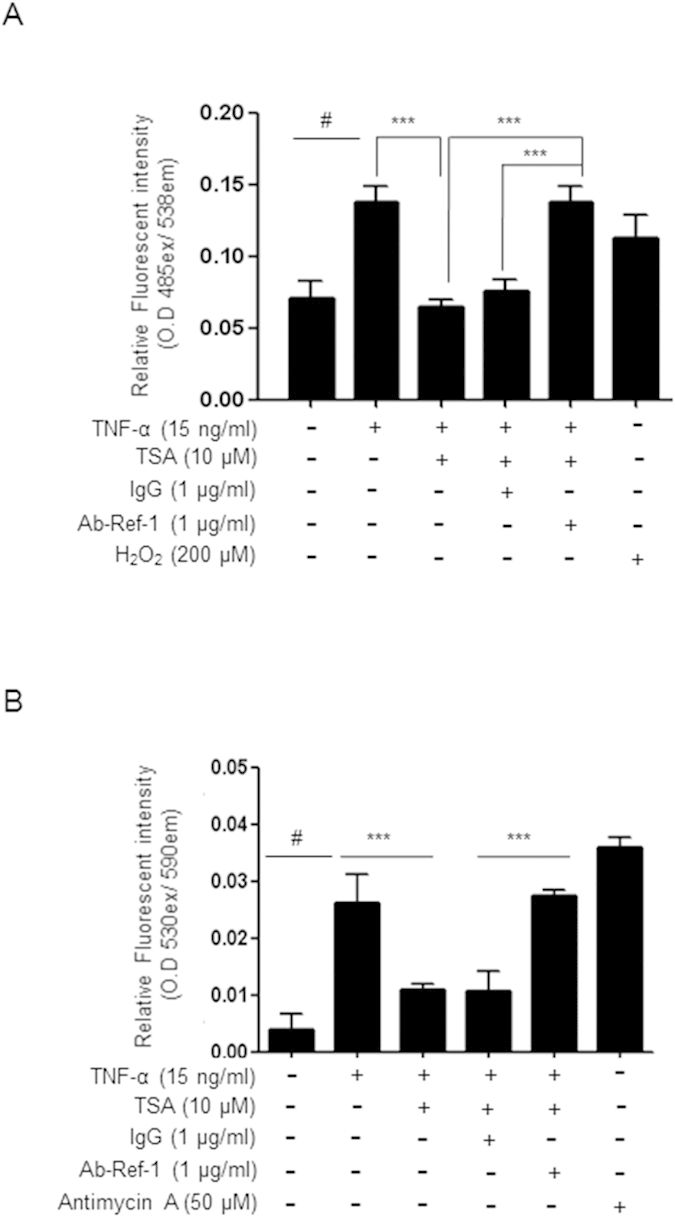
Treatment of neutralizing anti-APE1/Ref-1 prevented TSA-mediated suppression of ROS generation in TNF-α-stimulated HUVECs. (**A**,**B**) Intracellular or mitochondrial reactive oxygen species (ROS) generation was analyzed using DCFDA or MitoSOX fluorescent probes, respectively. Human umbilical vein endothelial cells were pretreated with TSA (10 μM), IgG (1 μg/ml), or anti-APE1/Ref-1(1 μg/ml) and then stimulated with TNF-α for 6 h. Hydrogen peroxide or antimycin A were used as a positive control, respectively. Columns, mean (n = 3); bars, SE. ***P < 0.001, significantly different from TSA/TNF-α- or IgG/TSA/TNF-α-treated cells; ^#^P < 0.01 significantly different from only untreated control cells by one-way ANOVA followed by Dunnett’s tests. Data shown are representative of replicate experiments with similar results.

Intracellular ROS generation is involved in inflammatory signaling in TNF-α-stimulated HUVECs^24^. Using a DCFDA fluorescent probe to examine intracellular ROS levels in TNF-α-stimulated endothelial cells, we detected the maximum intracellular ROS level at 6 h, at which point ROS levels were almost 2-fold higher in treated cells than in untreated control cells (Fig. 4A). The elevated ROS level in response to TNF-α decreased dramatically to basal levels after TSA-mediated acetylation, indicating an anti-inflammatory effect of TSA via inhibition of ROS production in TNF-α-stimulated HUVECs. To confirm whether secreted APE1/Ref-1 in response to TSA-mediated acetylation also affects ROS generation in TNF-α-stimulated HUVECs, we determined the effect of a neutralizing antibody on intracellular ROS levels. Similar to the observations for VCAM-1 expression, intracellular ROS generation increased after anti-APE1/Ref-1 antibody treatment, while TSA treatment inhibited ROS production in TNF-α-stimulated endothelial cells. IgG treatment did not affect the suppression of ROS generation in response to secreted APE1/Ref-1 in TNF-α-stimulated endothelial cells. These results indicated that secreted APE1/Ref-1 in response to TSA-mediated acetylation induces the inhibition of intracellular ROS generation, and the anti-inflammatory effect of secreted APE1/Ref-1 was almost completely neutralized by preventing its action via an anti-APE1/Ref-1 antibody.

Additionally, a change in mitochondrial ROS generation in response to TSA-mediated acetylation was observed using the mitochondrial superoxide indicator, MitoSox in TNF-α-stimulated endothelial cells. As shown in Fig. 4B, the increase in mitochondrial ROS by TNF-α-stimulation was decreased by 60% after treatment with TSA. However, the inhibited mitochondrial ROS generation was almost fully recovered in the presence of anti-APE1/Ref-1, confirming the functional role of secretory APE1/Ref-1 in anti-inflammatory signaling. In contrast, changes in the mitochondrial ROS level in response to TSA were maintained in cells that were pretreated with IgG. Collectively, these results proved that secreted APE1/Ref-1, but not acetylated APE1/Ref-1, is critical in the regulation of the initial inflammatory signal cascade via the binding of TNF-α to TNFR1.

### Recombinant human APE1/Ref-1 caused a conformational change in TNFR1, affecting its reducing ability

**Figure 5:**
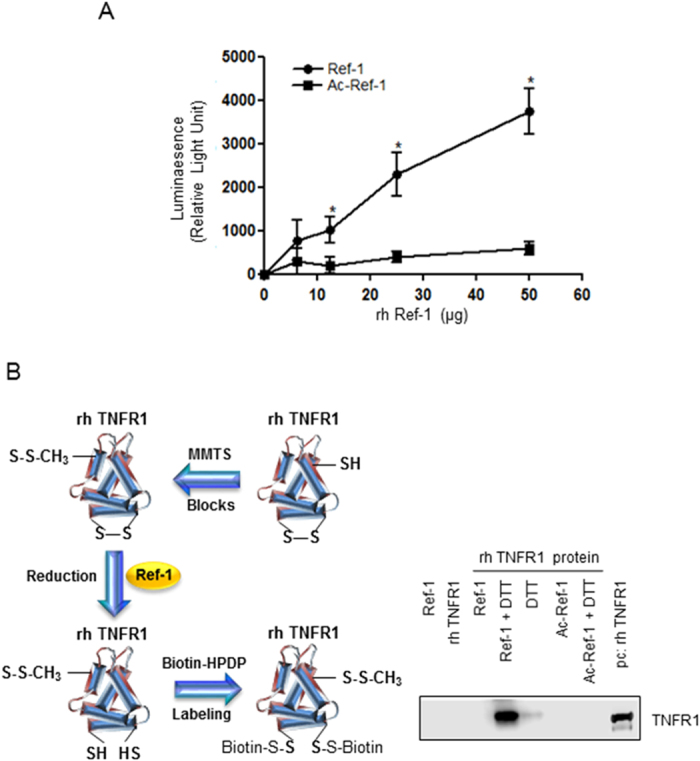
Recombinant human APE1/Ref-1 with reducing activity caused conformational change in TNF-α receptor via thiol-disulfide exchange. Reduction activity of recombinant human APE1/Ref-1 (rh APE1/Ref-1) was analyzed using biochemical reducing activity and a modified biotin-switch assay as described in the supplementary methods. (**A**) rh APE1/Ref-1 in a dose dependent manner was reacted with a reductase substrate, proluciferin, generating luminescent light. Mean (n = 5), SE. *P < 0.01, significantly different compared with control by one-way ANOVA followed by Dunnett’s tests (**B**) rh APE1/Ref-1 or rh Ac-APE1/Ref-1 in phosphate-buffered saline containing dithiothreitol (DTT*) was reacted with rh TNFR1 as shown in the schematic diagram of the modified biotin switch assay. DTT* was used for stable rh APE1/Ref-1 structure. The samples were resolved using PAGE without denaturation and only the reduced form of rh TNFR1 was detected by anti-TNFR antibody. A reducing agent, DTT, was used as a positive control. Note that acetylated APE1/Ref-1 (Ac-APE1) did not induce the reduction of rh TNFR1. Lane 1: APE1/Ref-1 (5 μg), Lane 2: TNFR1 (1 μg), Lane 3: APE1/Ref-1 (5 μg) + TNFR1 (1 μg), Lane 4: APE1/Ref-1 (5 μg) + DTT (10 mM), Lane 5: DTT (10 mM), Lane 6: Acetylated APE1/Ref-1 (5 μg), Lane 7: Acetylated APE1/Ref-1 + DTT (10 mM), Lane 8: TNFR1 (1 μg) as a positive control. These experiments were replicated three times with similar results. Representative blots are shown.

Next, we raised the question of whether secreted APE1/Ref-1 showed a redox function after deacetylation, leading to the regulation of TNFR1-mediated inflammatory signaling. We determined the redox activity of recombinant human APE1/Ref-1 (rh APE1/Ref-1) by measuring luminescent intensity, which was generated by luciferin formation after substrate reduction and reacted with luciferase, in a mixture containing the reductase substrate proluciferin and reductase rh APE1/Ref-1. The redox activity was proportional to the concentration of rh APE1/Ref-1 (Fig. 5A). Moreover, in the mixture containing rh Ac-APE1/Ref-1, the luminescent intensity was not statistically different from that of the control mixture, without reductase rh APE1/Ref-1.

Based on the observation that rh APE1/Ref-1, but not rh Ac-APE1/Ref-1, increased luminescent intensity, indicating reductive activity, we hypothesized that secreted APE1/Ref-1 can reduce other oxidized proteins, para- and autocrinally, in the culture supernatant after secretion. To evaluate the role of extracellular secreted APE1/Ref-1, we observed whether rh APE1/Ref-1 directly reduced rh TNFR1 containing 12 disulfide bonds via oxidization of free thiol groups. We performed a modified biotin-switch assay using recombinant TNFR1 as a substrate. As shown in Fig. 5B, rh APE1/Ref-1, which maintained its reduced form in the presence of 1 mM dithiothreitol (DTT), affected the reduction of TNFR1 as evidenced by S-biotinylation and binding to streptavidin-conjugated beads. Both rh APE1/Ref-1 and DTT caused a reduction in TNFR1 and the reducing activity of rh APE1/Ref-1 was higher than that of the reducing agent DTT. Neither oxidized rh APE1/Ref-1 nor Ac-APE1/Ref-1 even in the presence of DTT resulted in a conformational change of rh TNFR1. The results of the thiol-disulfide exchange reactions for TNFR1 strongly indicate that APE1/Ref-1, but not the acetylated form after secretion can induce the reduction of TNFR1. This suggests a conformational change in the extracellular domain and regulation of TNF-α-stimulated inflammation.

### Secreted APE1/Ref-1 suppressed activation of p66^shc^ and p38 in TNF-α/TNFR1 inflammation signaling, and this activation was recovered by treatment with a neutralizing APE1/Ref-1 antibody

**Figure 6:**
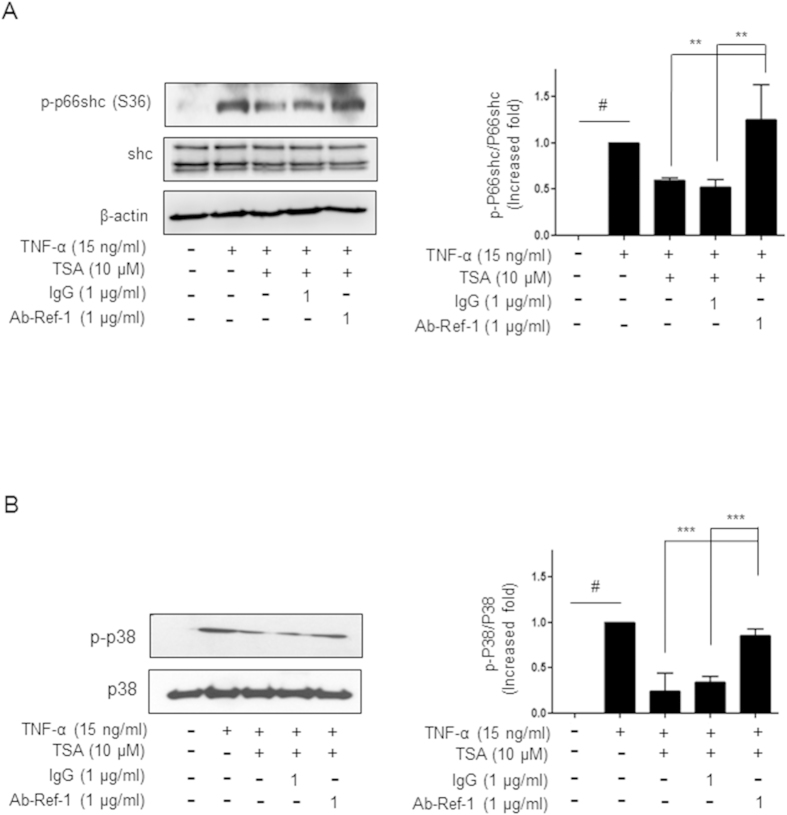
Treatment with neutralizing anti-APE1/Ref-1 caused re-activation of suppressed p66^shc^ and p38 MAPK in TNF-α-stimulated HUVECs after TSA-mediated acetylation. (**A**,**B**) Human umbilical vein endothelial cells were pretreated with only TSA (10 μM), IgG (1 μg/ml), or anti-APE1/Ref-1(1 μg/ml)/TSA and then stimulated with TNF-α. HUVEC lysates were obtained and subjected to immunoblotting. (**A**) Immunoblotting for phospho-p66^shc^ (**B**) Immunoblotting for phospho-p38 MAPK. The blots were stripped and reprobed with anti-p66^shc^, -p38, or -β-actin antibodies to ensure equal protein loading. Immunoblotting for each protein was performed two or more times by using independently prepared lysates and similar results were obtained. Fold-changes in phosphorylated vs. total protein or the levels of apoptosis markers relative to the control are shown for each time point. *Columns*, mean (n = 2–3); *bars*, SE. **P < 0.01, significantly different from TSA/TNF-α- or IgG/TSA/TNF-α-treated cells; ^#^P < 0.01, significantly different from only untreated control cells by one-way ANOVA followed by Dunnett’s tests. These experiments were replicated with similar results. Representative blots are shown.

To gain insight into the mechanism of secreted APE1/Ref-1-mediated suppression of TNF-α/TNFR1 inflammatory signaling, the involvement of p66^Shc^ and MAPKs was investigated. As shown in Fig. 6A,B, the phosphorylation level of p66^shc^ on serine 36 residue increased in TNF-α-treated endothelial cells and then considerably decreased by 50% after TSA treatment. Consistent with the results for ROS generation, suppressed p66^Shc^ was almost completely reactivated after treatment with a neutralizing APE1/Ref-1 antibody, to a level similar to that of TNF-α treated cells, although immunoglobulin did not affect the reactivation of p66^shc^. Additionally, the contribution of MAPK activation to TNF-α-induced Ser-phosphorylation of p66^Shc^ was studied. When TNF-α- and TSA-treated cells were incubated with neutralizing APE1/Ref-1 antibody, the suppressed phosphorylation of p66^shc^ increased significantly (p < 0.001 vs. TNF-α-/TSA-treated cells) as shown in Fig. 6A,B. By contrast, treatment with the neutralizing antibody in JNK or ERK involvement to phosphorylation p66^shc^ in TNF-α/TNFR1 inflammatory signaling did not observed (data not shown). Thus, these results suggest that p66^Shc^ and p38, but not JNK or ERK, are involved in anti-inflammatory signals via secreted APE1/Ref-1 in TNF-α-stimulated cells.

## Discussion

An understanding of the function of extracellular APE1/Ref-1 is necessary for its application as an anti-inflammatory agent and to establish clinical trials to support a mechanism-based approach to treat vascular inflammatory disease. The present study provides novel evidence for the role of the auto- or paracrine secretory protein in inflammation and indicates that the anti-inflammatory regulation of secreted APE1/Ref-1 in TNF-α-stimulated endothelial cells after TSA treatment is tightly linked to the inactivation of TNF-α/TNFR1 signaling. This conclusion is based on the following observations: (a) TSA-mediated acetylation causes the secretion of Ac-APE1/Ref-1 in TNF-α-stimulated endothelial cells, which show a substantial decrease in VCAM-1 expression; (b) secreted Ac-APE1/Ref-1 was rapidly deacetylated; (c) not only the suppression of VCAM-1 expression and ROS generation, but also the inactivation of p66^Shc^ and p38 MAPK in TSA-mediated acetylated cells were recovered by treatment with a neutralizing anti-APE1/Ref-1 antibody; (d) rh APE1/Ref-1 caused a conformational change in the extracellular domain of TNFR1, showing substantial reducing ability in biochemical assays. Because the acetylation-induced anti-inflammatory effects in TNF-α-stimulated endothelial cells was inhibited by pretreatment with the neutralizing antibody, it is reasonable to conclude that secreted APE1/Ref-1, but not the acetylated form, initially regulates inflammatory signals via disruption of TNFR1, preventing binding with TNF-α.

Acetylation, a type of post-translational modification, is considered an important mechanism in the regulation of gene expression via activation of nuclear histone proteins; it regulates the functions of multiple cytoplasmic proteins involved in cell survival, proliferation, and death, as well as disease-related intracellular signaling. Cellular acetylation increases rapidly via HDACi administration. HDAC inhibitors are potential chemotherapeutic agents for cancer and inflammatory diseases^26^,^27^,^28^. Interestingly, previous studies have shown that TSA treatment induces remarkable regulation of TNF-α-induced VCAM-1 expression and thereby inhibits monocyte adhesion both *in vitro* and *in vivo*, even though the molecular basis for this effect is unclear. Moreover, acetylation at a specific amino acid residue, i.e., lysine, and changes in the function of cellular proteins have also been reported, including changes in DNA-protein interactions, transcriptional activity, protein stability, and enzymatic activity^29^. Treatment with HDACi, which suppresses the removal of acetyl groups from lysine residues, causes intracellular acetylation and dramatic extracellular translocation of several proteins^30^. TSA-mediated acetylation induces the secretion of APE1/Ref-1, this effect is dependent on K6/K7 acetylation in HEK293 cells^31^. Acetylation of hsp90α by treatment with a pan-HDAC inhibitor causes hyperacetylation and extracellular secretion, increasing *in vitro* tumor cell invasiveness^32^. Inhibition of HDAC-1 and −4 in hepatocytes causes an increase in HMGB-1 release, indicating that acetylation is critical for active HMGB-1 release^33^.

Our observation that APE1/Ref-1 was secreted in TNF-α-stimulated endothelial cells after TSA-mediated hyperacetylation strongly supports the functional role of extracellular APE1/Ref-1. The secreted and acetylated APE1/Ref-1 showed no redox activity owing to conformational changes via acetylation according to the results of biochemical assays. However, the Ac-APE1/Ref-1 could be rapidly converted to its native form by removing the acetyl moiety from the ε-amino groups of the protein substrate lysine residue, indicating the recovery of reducing activity. APE1/Ref-1 contains two cysteine residues (cysteines 65 and 93) in its redox active site^34^. A sulfhydryl switch mechanism in which cysteine 65/93 of APE1/Ref-1 interacts with the conserved cysteines within other proteins also suggests the possible role of deacetylated APE1/Ref-1 as a reductant^35^. For example, thioredoxin (Trx) reduces oxidized cysteine groups via an interaction with the redox-active center using SH groups as reducing equivalents^36^. The oxidized, Trx-containing disulfide bond is then reduced by thioredoxin reductase (TrxR) and NADPH. Further studies are needed to explore the existence of associated molecule, like the Trx system, involved in the recognition of secreted Ac-APE1/Ref-1 or nonenzymatic dissociation of acetyl groups, promoting conversion to APE1/Ref-1 with reducing activity. Nonetheless, it is clear that secreted APE1/Ref-1, but not Ac-APE1/Ref-1, is negatively correlated with inflammatory signaling in TNF-α-stimulated endothelial cells.

In TNF-α-stimulated inflammation, TNFR1 is a potentially selective substrate for secreted APE1/Ref-1. TNFR1 is characterized by the ability to bind TNF-α via extracellular CRDs; the receptor itself is comprised of a chain of four CRDs, each of which is stabilized by three pairs of disulfide-linked cysteine residues^25^. To initiate intracellular inflammatory signal transduction, homotrimeric TNF-α directly binds to CRDs of the receptor molecule, leading to signal-competent multimerization of unligated TNFRs^25^. Interestingly, there is some evidence showing that some disulfide bonds at the cell surface existing mainly in an oxidized redox state are labile, affecting activity^37^. For example, representative reducing agents, DTT or *N*-acetylcysteine can cleave disulfide bonds, resulting in a change in protein structure, and hence a change in function^37^. In addition, TRX can modulate the activity of cluster of differentiation 30, a member of the TNFR family, via the selective reduction of a disulfide bond, despite their high content of disulfide bonds^37^. Interferon gamma-inducible lysosomal thiol reductase, which is secreted from circulating macrophages after exposure to bacterial lipopolysaccharides (LPS), plays a role in major histocompatibility complex class II-restricted processing and the presentation of antigens that contain disulfide bonds. Because, rh APE1/Ref-1 causes the reduction of disulfide bonds in the extracellular domain of rh TNFR1, and treatment with neutralizing anti-APE1/Ref-1 antibody rapidly prevents acetylation-mediated anti-inflammatory signals in TNF-α-stimulated endothelial cells, it seems reasonable to postulate that down regulation of ROS, p66^shc^/MAPK, and VCAM-1 expression are induced by secreted APE1/Ref-1, either auto- or paracrinally. However, further studies are needed to systematically explore this possibility, including the identification of molecules that promote the interaction with the substrate TNFR1.

It is plausible that multiple disulfide bonds in the extracellular domain of other transmembrane receptors can be affected by reducing proteins. Cytokines and biochemical mediators released during inflammation intensify and propagate the inflammatory response. These mediators can act systemically and activate other receptors simultaneously. Based on our studies, inflammatory reactions stimulated by cytokines through the IL-1 receptor or the Toll-like receptor (each has 5 disulfide bonds in the extracellular domain) were effectively attenuated by exposure to APE1/Ref-1 (Supplementary Figures 4 and 5). In accordance with TNF-α/TNFR regulation, the IL-1 or LPS-stimulated inflammatory signal was also inhibited by reduction of disulfide bonds, implying a broad-spectrum reducing effect of APE1/Ref-1.

In conclusion, the present study offers novel insight into the mechanisms of hyperacetylation-mediated anti-inflammation signals in human TNF-α-endothelial cells and shows a strong relationship between secreted APE1/Ref-1, but not Ac-APE1/Ref-1, and TNF-α/TNFR1 and the inflammatory process. Based on these observations, we propose that rh APE1/Ref-1 with reducing activity may be a useful therapeutic agent for the treatment of cytokine responses involved in vascular inflammation. Clarifying the mechanisms of the post-translational modification of secreted APE1/Ref-1 may provide specific markers for vascular diseases and facilitate the development of a vascular inflammation inhibitor.

## Methods

### Cell culture

HUVECs were cultured in endothelial growth medium as previously reported^38^. Cells (passages 4–8) were maintained at 37 °C in a humidified atmosphere of 5% CO_2_.

### Adenoviral infection

Adenoviruses encoding β-galactosidase (Adβ-gal), full-length APE1/Ref-1 (AdAPE1/Ref-1), and HDAC3 (AdHDAC3) were prepared^22^ and HUVECs were infected at the indicated multiplicity of infection (MOI; particle forming units per cell) of the specified adenovirus for 24 h. The virus-containing medium was replaced for remove unbound virus removal and the cells were incubated further to overexpress each protein for 24 h.

### Identification of secreted Ac-APE1/Ref-1

HUVECs were pretreated with TSA for the indicated time periods and concentrations followed by treatment with 15 ng/ml TNF-α for 12 h. The secreted acetylated form, APE1/Ref-1 in the culture supernatant was chemically pulled-down^23^ or immunoprecipitated using the anti-APE1/Ref-1 antibody or anti-acetyl-lysine antibody as previously reported^23^. In some experiments, anti-acetyl lysine agarose beads were used for the rapid and specific pull-down of acetylated proteins. See the Supplementary Materials and Methods section for details.

### ROS generation

The effects of secreted APE1/Ref-1 on intracellular ROS generation or mitochondrial superoxide production in TNF-α-stimulated endothelial cells were analyzed as described previously with modifications^38^. Cells were pretreated with IgG or anti-APE1/Ref-1 before exposure to TNF-α and TSA. Briefly, intracellular ROS levels were measured using a dichlorofluorescin diacetate (DCFDA) fluorescent probe and the mitochondrial superoxide indicator MitoSOX. See the Supplementary Materials and Methods.

### Quantitative analysis of secreted APE1/Ref-1

A sandwich enzyme-linked immunosorbent assay (ELISA) was used to quantify the level of secreted APE1/Ref-1 in the culture supernatant as previously described^21^.

### rh APE1/Ref-1 activity assay

Purified rh APE1/Ref-1 was prepared as previously reported^20^, and the rh APE1/Ref-1 was maintained in the presence of 1 mM DTT and stored in liquid nitrogen until use. The reducing activity of rh APE1/Ref-1 was biochemically measured with the NAD(P)H-Glo Detection System (Promega, Madison, WI, USA) according to the manufacturer’s protocol with modifications. Additionally, the activity of rh APE1/Ref-1 was confirmed using a biotin-switch with rh TNFR1 as a substrate, as described in the Supplementary Materials and Methods.

### Western blot analysis

Whole cell lysates were prepared and subjected to SDS-PAGE (sodium dodecyl sulfate polyacrylamide gel electrophoresis) and immunoblotting as described in the Supplementary Materials and Methods.

### Statistical analysis

All statistical tests were implemented in GraphPad Prism v.5.01 (La Jolla, CA, USA) software. Statistical significance in the differences in measured variables between the control and treated groups was determined by a one-way analysis of variance (ANOVA) followed by Dunnett’s or Bonferroni’s multiple comparison tests. Differences were considered significant at P < 0.05.

## Additional Information

**How to cite this article**: Park, M. S. *et al.* Secreted APE1/Ref-1 inhibits TNF-α-stimulated endothelial inflammation via thiol-disulfide exchange in TNF receptor. *Sci. Rep.* **6**, 23015; doi: 10.1038/srep23015 (2016).

## Supplementary Material

Supplementary Information
