# Supplementary material for: Secreted APE1/Ref-1 inhibits TNF-α-stimulated endothelial inflammation via thiol-disulfide exchange in TNF receptor
Source: Sci Rep. 2016 Mar 11;6:23015. doi: 10.1038/srep23015 (PMC4786854; doi:10.1038/srep23015)
Supplement: Supplementary Information [file srep23015-s1.doc]

**Supplementary Information**

**Secreted APE1/Ref-1 inhibits TNF-α-stimulated endothelial inflammation via thiol-disulfide exchange in TNF receptor**

Myoung Soo Park^*^, Sunga Choi^*^, Yu Ran Lee, Hee Kyoung Joo, Gun Kang, Cuk-Seong Kim, Soo Jin Kim, Sang Do Lee, Byeong Hwa Jeon^#^

Infectious Signaling Network Research Center and Research Institute for Medical Sciences, Department of Physiology, School of Medicine, Chungnam National University, Daejeon, 301-747, Republic of KOREA.

Address correspondence to: Byeong Hwa Jeon, MD, Ph.D. Department of Physiology, School of Medicine, Chungnam National University. 266 Munhwa-ro, Jung-gu, Daejeon, 301-747, Korea Phone: +82-42-580-8214; Fax :+82-42-585-8440, E-mail : bhjeon@cnu.ac.kr

**
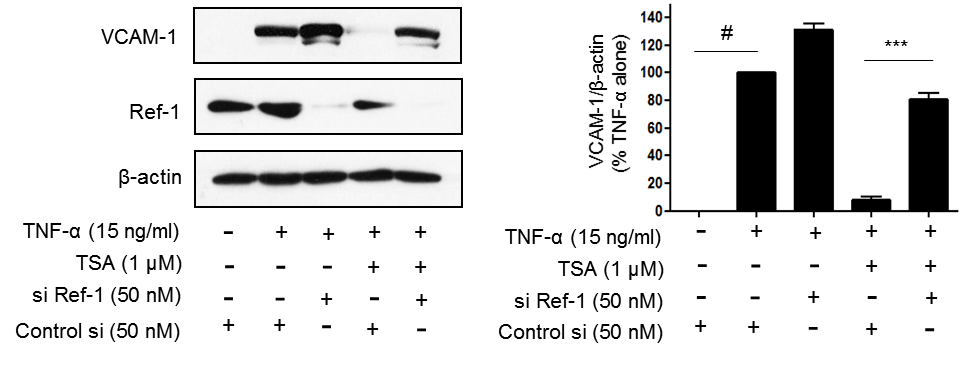
**

**Supplementary Figure 1. TSA treatment did not regulate VCAM-1 expression in TNF-α-stimulated HUVECs following transfection with APE1/Ref-1 siRNA.**

Effect of trichostatin A (TSA)-mediated acetylation on VCAM-1 expression in TNF-α-stimulated human umbilical vein endothelial cells (HUVECs), which were transfected with APE1/Ref-1-specific siRNA. Cells were treated with TSA (1 μM) in the presence or absence of TNF-α (15 ng/ml). The blots were stripped and reprobed with anti-APE1/Ref-1 to confirm APE1/Ref-1 knock-down or actin antibodies to ensure protein loading. Representative blots are shown. The bar graph shows densitometry quantification of the western blot data. The data are represented as % densitometry values of TNF-α-induced VCAM-1 expression. *Columns*, mean (n = 3); *bars*, SE. ***, P < 0.001, significantly different from TSA treated-, TNF-α-stimulated cells with down-regulation of APE1/Ref-1; #, P < 0.01, significantly different from untreated control cells based on a one-way analysis of variance (ANOVA) followed by Dunnett’s test. Similar results were observed in replicate experiments.

**
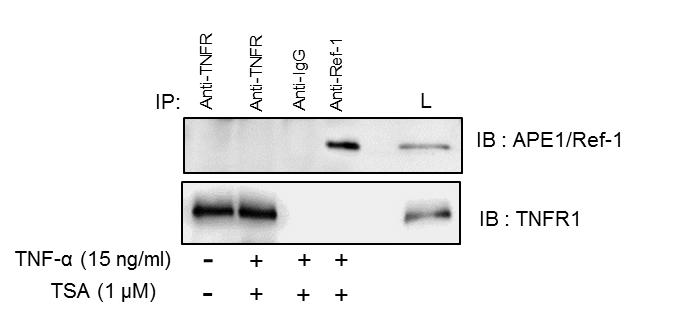
**

**Supplementary Figure 2. Secreted APE1/Ref-1 in response to TSA did not bind directly with tumor necrosis factor receptor (TNFR) 1 in TNF-α-stimulated HUVECs.**

After cells were treated with TSA (1 μM) for 1 h, followed by treatment with 15 ng/ml TNF-α for 1 h, the TNFR1 was immunochemically precipitated. The TNFR1 was reacted with secreted APE1/Ref-1 in the culture supernatant. The binding complex was confirmed by immunoblotting using anti-APE1/Ref-1 antibody. The presence of APE1/Ref-1 in the culture supernatant was confirmed by immunoprecipitation using an anti-APE1/Ref-1 antibody and an anti-TNFR1 antibody was used to confirm the presence of an equal amount of TNFR1 protein. L; cell lysate was used as a positive control. Replicate experiments produced similar results. Representative blots are shown.

**
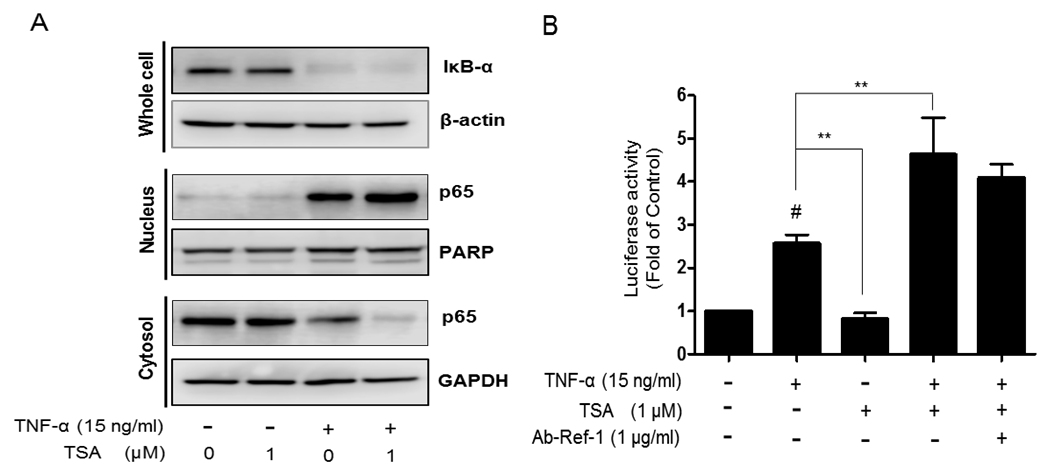
**

**Supplementary Figure 3.** **TSA treatment did not affect the NF-κB signaling pathway in TNF-α-stimulated HUVECs.**

A. After cells were treated with TSA (1 μM) for 1 h, followed by treatment with 15 ng/ml TNF-α for 0.5 h, cell lysates were obtained. A p65-NF-κB immunoblot was performed using fractionated nuclear and cytosolic cell lysates. PARP and GAPDH were used as loading controls for the nuclear and cytosolic proteins, respectively. Immunoblotting for IκB-α was also performed using whole cell lysate. The blot was stripped and reprobed with an anti-β-actin antibody to correct for differences in protein loading. Similar results were observed in replicate experiments. B. Transcriptional activity of NF-κB in HUVECs stimulated with TNF-α for 6 h, following treatment with 1 μM TSA, was determined using a luciferase reporter gene assay. *Columns*, mean (n = 3); *bars*, SE. **, P < 0.01, significantly different from TNF-α-treated control cells; #, P < 0.01, significantly different from untreated control cells, as determined by one-way ANOVA followed by Dunnett’s test. Similar results were observed in replicate experiments.


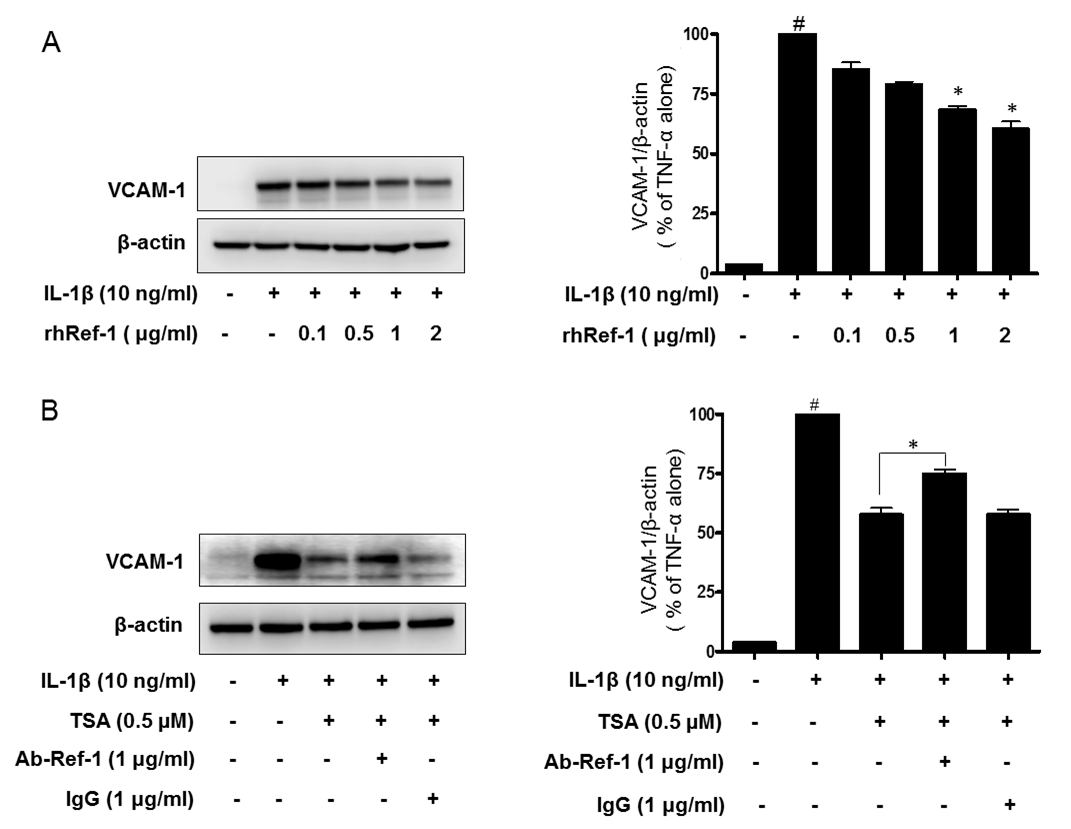


**Supplementary Figure 4. Secreted APE1/Ref-1 inhibited VCAM-1 expression in interleukin-1β-stimulated HUVECs.**

A. rh APE1/Ref-1 inhibited IL-1β-induced VCAM-1 expression. rh APE1/Ref-1 was pretreated for 0.5 h at the indicated concentrations. B. Neutralization of APE1/Ref-1 inhibited TSA-mediated suppression of IL-1β–induced VCAM-1 expression. Anti-APE1/Ref-1 antibody or anti-rabbit IgG was pretreated for 0.5 h and then 1 μM TSA was added before stimulation of human umbilical vein endothelial cells (HUVECs) with interleukin-1β (IL-1β). HUVECs lysates were obtained and immunoblotting for VCAM-1 was performed. The blots were stripped and reprobed with anti-β-actin antibody to ensure equal protein loading. Immunoblotting for each protein was performed three times using independently prepared lysates and similar results were obtained. Columns, mean (n = 3); bars, SE. *, P < 0.05, significantly different from IL-1β/TSA-treated cells; #, P < 0.01 significantly different from untreated control cells by one-way ANOVA followed by Dunnett’s tests.

**
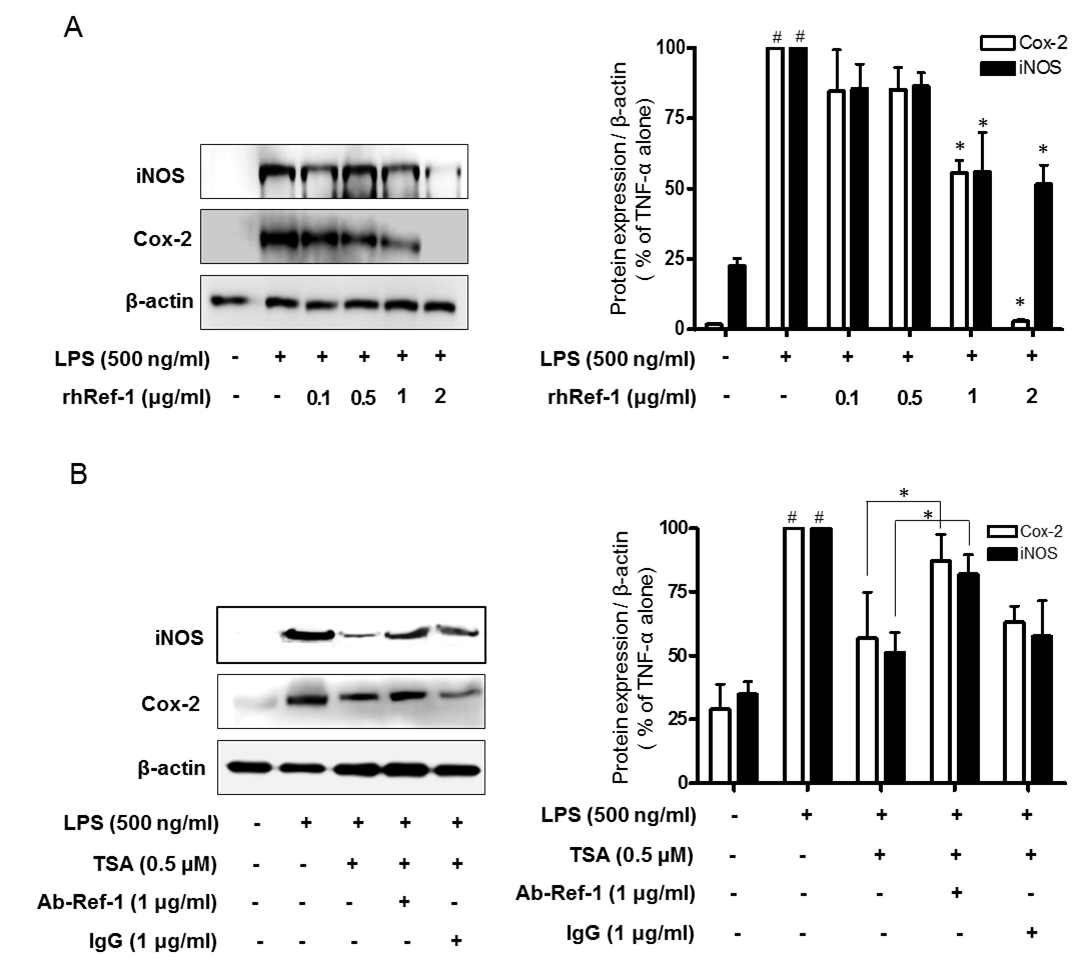

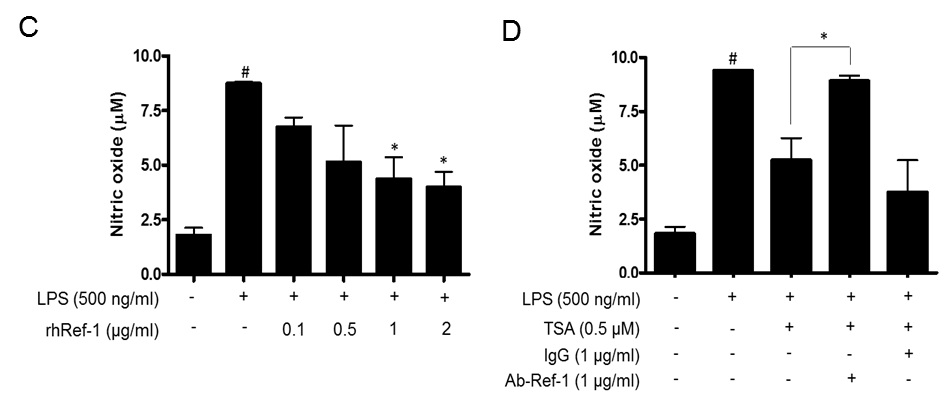
**

**Supplementary Figure 5. Secreted APE1/Ref-1 inhibited iNOS or COX-2 expression in lipopolysaccharide-stimulated RAW 264.7 macrophage cells.**

A. rh APE1/Ref-1 inhibited LPS-induced VCAM-1 expression. rh APE1/Ref-1 was pretreated for 0.5 h at the indicated concentrations. B. Neutralization of APE1/Ref-1 inhibited TSA-mediated suppression on LPS-induced VCAM-1 expression. Anti-APE1/Ref-1 antibody or anti rabbit IgG was pretreated for 0.5 h and then 1 μM TSA was added before stimulation of murine RAW 264.7 macrophage cells with lipopolysaccharide (LPS). RAW 264.7 macrophage cells lysates were obtained and immunoblotting for inducible nitric oxide synthase and cyclooxygenase-2 was performed. The blots were stripped and reprobed with anti-β-actin antibody to ensure equal protein loading. Immunoblotting for each protein was performed three times using independently prepared lysates and similar results were obtained. C and D. Culture supernatant of RAW 264.7 macrophage cells was collected and analyzed for nitric oxide levels. Columns, mean (n = 3); bars, SE. *, P < 0.05, significantly different from LPS/TSA-treated cells; #, P < 0.01 significantly different from untreated control cells by one-way ANOVA followed by Dunnett’s test.

**Materials and Methods**

**Reagents**

Human umbilical vein endothelial cells (HUVECs) and endothelial growth medium (EGM-2, were purchased from Clonetics (Baltimore, MD, USA) and Lonza (Walkersville, MD, USA), respectively. The murine macrophage cell line RAW264.7 and Dulbecco’s modified Eagle’s medium were purchased from the American Type Culture Collection (Manassas, VA, USA) and Welgene (Daegu, South Korea), respectively. Human tumor necrosis factor–α (TNF-α), trichostatin A (TSA), methanethiosulfonate (MMTS), dimethylformamide (DMF), ascorbic acid, hydrogen peroxide, antimycin A, and LPS (*Escherichia coli* O26:B6) were purchased from Sigma-Aldrich (St. Louis, MO, USA). Recombinant human IL-1β was purchased from R&D System (Minneapolis, MN, USA). EZ-Link Biotin-HPDP, ZEBA columns, and recombinant tumor necrosis factor receptor (TNFR) were purchased from Thermo (Thermo Inc., San Jose, CA, USA) and Abcam (Cambridge, UK). The NE-PER nuclear and cytoplasmic extraction kit and the dual luciferase assay kit were purchased from Thermo (Thermo Inc., San Jose, CA, USA) and Promega (Madison, WI, USA), respectively. Human APE1/Ref-1-specific siRNA duplexes (cat. #1006541) and non-specific siRNA were purchased from Bioneer Co. (Daejeon, South Korea). Streptavidin-Sepharose conjugate 4B was purchased from Invitrogen-Life Technologies (Carlsbad, CA, USA). Antibodies against VCAM-1, p65-NF-κB, IκB-α, poly (ADP-ribose) polymerase (PARP), GAPDH, inducible nitric oxide synthase (iNOS), cyclooxygenase 2 (COX-2) and rabbit IgG were obtained from Santa Cruz Biotechnology (Santa Cruz, CA, USA); antibodies against FLAG and β-actin were obtained from Sigma-Aldrich; anti-acetyl-lysine antibody was obtained from Cell Signaling (Danvers, MA, USA); monoclonal antibody against APE1/Ref-1 (80–100 N-terminal amino acids) was purchased from Novus Biologicals (Littleton, CO, USA). Anti-acetyl lysine agarose beads were obtained from ImmuneChem Pharmaceuticals (Burnaby, Canada). A polyclonal APE1/Ref-1 antibody was generated by immunizing rabbits with recombinant human APE1/Ref-1 (rh APE1/Ref-1)^1^ and purification using affinity chromatography.

**Methods**

**Immunoprecipitation**

HUVECs were grown to confluence on a 60-mm plate (5 × 10^5^ cells/wells) and then pretreated with 1 μM TSA for 1 h followed by treatment with 15 ng/ml TNF-α for indicated time. The culture supernatant was carefully collected and centrifuged at 1,200 rpm (150 *g*) for 3 min. The separated supernatant without perturbing precipitates was centrifuged second time at 1,500 rpm (850 *g*) for 3 min to remove contaminants from cell debris thoroughly. The supernatant was immunoreacted with anti-acetyl-lysine antibody. Protein A/G agarose beads were then added to each sample and incubated at 4°C on a rotary shaker. In some experiments, HUVECs were pretreated with 1 μM TSA for 1 h followed by treatment with 15 ng/ml TNF-α for 1 h. After careful collection of the culture supernatant, cells were harvested in 200 µl of lysis buffer. Cell lysates were prepared by centrifugation at 12,000 rpm for 15 min. The TNFR1 in the cell lysate was pulled down using anti-TNFR1 antibody. Protein A/G agarose beads were then added to each sample and further incubated with the culture supernatant. The immunocomplex was collected by centrifugation at 3,000 rpm for 3 min and washed three times with buffer (50 mM Tris-HCl pH 7.5, 1% TX-100, 5% glycerol, 10 mM butyrate, 10 mM nicotinamide, 5 μM TSA, protease and phosphatase inhibitors). Each sample was mixed with a sample buffer, boiled, and subjected to 12% sodium dodecyl sulfate polyacrylamide gel electrophoresis (SDS-PAGE), which was followed by immunoblotting using monoclonal anti-APE1/Ref-1 or anti-acetyl-lysine antibody. Anti-acetyl-lysine antibody is specific to acetylated proteins.

**ROS generation**

HUVECs were pre-incubated with IgG or anti-APE1/Ref-1 for 1 h and treated with TSA as the indicated concentrations and times. After stimulation with 15 ng/ml TNF-α for 1 h, the cells were stained with 5 μM H_2_DCFDA for 30 min at 37°C. Cells were treated with 100 μM H_2_O_2_ without TNF-α as a positive control. The cells were collected and fluorescence was analyzed using a fluorometer (Thermo Scientific, Rockford, IL, USA) with a 485 ex/530 em filter set^2^. Mitochondrial superoxide production was also measured using MitoSOX red, a mitochondria-specific dye (Molecular Probes, Eugene, OR, USA). After exposure to TNF-α for 6 h, the cells were incubated with 5 μM MitoSOX red for 10 min. The relative fluorescence intensity was measured at 530 em/590 em^3^. Some cells were treated with 100 μM H_2_O_2_ or 50 μM antimycin A and used as a positive control for intracellular or mitochondrial ROS, respectively.

**Nitric oxide production**

The level of nitric oxide (NO) in the culture supernatant was measured using a colorimetric assay kit (Abcam) according to the manufacturer’s protocol. In brief, 12 h after LPS treatment, phenol-free media from cells was collected and deproteinized using a 10 kDa centrifugal filter. The samples and standard (1–100 μM) were exposed to nitrate reductase and cofactors for 1 h at room temperature to transform nitrate to nitrite. Following addition of the enhancer, Griess reaction reagents were applied to convert nitrite to a purple azo chromophore compound and developed for 10 min. The absorbance was measured at 540 nm using a microplate reader. Absorbance of samples was adjusted to control for background levels determined in the phenol-free culture media.

**Biochemical assay of rh APE1/Ref-1 activity**

rh APE1/Ref-1 activity was analyzed using a modified NAD(P)H-Glo Detection System (Promega, Madison, WI, USA) containing a reductase substrate and luciferin detection reagent. Briefly, 50 μg of rh APE1/Ref-1 as a reductase was prepared by 2-fold serial dilution in phosphate-buffered saline containing 50 μM DTT (final concentration) for stable rh APE1/Ref-1 structure. Samples containing only 50 μM DTT were serially diluted and analyzed simultaneously. The intensity was subtracted from the luminescence of the respective rh APE1/Ref-1 value. In 96-well white luminometer-compatible plates, the rh APE1/Ref-1 reductase was reacted with reductase substrate with the proluciferin moiety and then converted to luciferin after reduction. The luciferin in the reaction mixture was detected by luciferase in the detection reagent, and the amount of light was measured using a luminometer. The intensity was proportional to the amount of rh APE1/Ref-1 in the sample. This assay was performed in triplicate, and the mean of the data points and the standard error of the means were calculated.

**Biotin switch assay for rh APE1/Ref-1**

One microgram of rh TNFR1 in HEN buffer (containing 250 mM HEPES, 1 mM EDTA, 0.1 mM neocuproine, pH 7.7) was incubated with 10 mM methyl methanethiosulfonate (MMTS) at 50°C for 20 min, and excess MMTS was removed by passing through the ZEBA spin column three times. For the reduction of the disulfide bonds in the extracellular domain of TNFR1, rh TNFR1 was reacted with 5 µg of rh APE1/Ref-1 or Ac-APE1/Ref-1 in the presence or absence of DTT. Samples containing only DTT were also prepared as positive controls. Samples containing only rh APE1/Ref-1 or rh TNFR1 without the respective enzyme or substrate were analyzed. Each sample was supplemented with labeling buffer containing 1 mM ascorbate and 4 mM N-[6-(biotinamido)hexyl]-3-pyridyldithio)-propionamide (biotin-HPDP) at room temperature with rotation for 1 h. Unreacted biotin-HPDP was removed using the Zeba spin column, and the buffer was changed with neutralization buffer (20 mM HEPES, 100 mM NaCl, 1 mM EDTA, 0.5% Triton X-100, pH 7.7). The biotinylated proteins were pulled-down with 30 μl of streptavidin-agarose overnight at 4°C. Pellets were then washed five times with neutralization buffer containing 0.6 M NaCl, eluted by SDS sample buffer without the reducing agent, and subjected to immunoblot analysis using the anti-TNFR-1 antibody.

**Immunoblot analysis**

Cultured HUVECs were harvested in 80 µl of lysis buffer (20 mM Tris-HCl, pH 7.5, 150 mM NaCl, 1 mM EGTA, 1% NP-40, 1% Na deoxycholate, 2.5 mM Na pyrophosphate, 1 mM Na3VO3, 1 mM β-glycerophosphate, and a protease inhibitor cocktail). Cell lysates were prepared by centrifugation at 12,000 rpm for 15 min, and the supernatant was collected. Proteins (30 µg) were separated by 10% SDS-PAGE and transferred to a PVDF (polyvinylidene fluoride) membrane. After blocking with 5% non-fat dry milk in TBS containing 0.05% Tween 20, the membrane was incubated with VCAM-1, phospho-p66^shc^, p66^shc^, iNOS, or COX-2 antibody for 18 h at 4°C. The membrane was treated with an appropriate peroxidase-conjugated secondary antibody, and the chemiluminescent signal was developed using the Super Signal West Pico or Femto Substrate (Pierce Biotechnology, Rockford, IL, USA). Each membrane was again reacted with anti-β-actin antibody to normalize loading.

**RNA interference of APE1/Ref-1**

RNA interference of APE1/Ref-1 was performed using human APE1/Ref-1 specific siRNA duplexes. For transfection, HUVECs were seeded in 6-well plates and transfected at 50% confluency with siRNA duplexes (50 nM) using lipofectamine 2000 (Invitrogen, Carlsbad, CA, USA) according to the manufacturer’s recommendations. Cells transfected with non-specific siRNA were used as controls for direct comparison. After 48 h of transfection, cells were pretreated with 1 μM TSA for 1 h followed by treatment with 15 ng/ml TNF-α for 12 h. Cells were collected and processed for analysis of VCAM-1 expression.

**Preparation of nuclear and cytosolic fractions**

Nuclear and cytosolic fractions were prepared from control and TNF-α/TSA co-treated HUVECs using a NE-PER nuclear and cytoplasmic extraction kit according to the manufacturer’s instructions. Briefly, the cells were lysed and centrifuged at 12,000 rpm for 10 min. The supernatant (cytosolic fraction) and nuclear pellet were collected. The nuclear pellet was re-suspended in nuclear buffer (20 mM HEPES, pH 7.9, 0.4 M NaCl, 1 mM EDTA, 10% glycerol, 1 mM DTT and protease inhibitors), vortexed, passed (15–20 times) through a 27-gauge needle, and centrifuged at 12,000 rpm for 20 min. The supernatant fractions were collected and used for immunoblotting of p65-NF-κB.

**Luciferase reporter assay**

HUVECs were plated at a density of 2 × 10^5^ cells/well in 6-well plates, allowed to attach overnight and transfected with 1 μg of total plasmid containing 0.99 μg/well NF-κB-luciferase reporter vector^4^ and 10 ng/well of pCMV-pRL internal control vector (Promega) using the transfection reagent, Effectene (Qiagen, Valencia, CA, USA). After transfection, the cells were pretreated with 1 μM TSA for 1 h, followed by treatment with 15 ng/ml TNF-α for 6 h, washed with ice-cold PBS, and harvested in reporter lysis buffer. After centrifugation, 20 μl of the supernatant fraction was used for measurement of dual luciferase activity using a luminometer. The luciferase activity was normalized against protein concentration and expressed as relative luciferase activity (a ratio of firefly luciferase to Renilla luciferase units).

**Reference**

1. Park, M. S. *et al.* Identification of plasma APE1/Ref-1 in lipopolysaccharide-induced endotoxemic rats: implication of serological biomarker for an endotoxemia. *Biochemical and biophysical research communications* **435**, 621-626 (2013).

2. Choi, S. *et al.* A standardized bamboo leaf extract inhibits monocyte adhesion to endothelial cells by modulating vascular cell adhesion protein-1. *Nutrition research and practice* **7**, 9-14 (2013).

3. Joo, H. K. *et al.* Peripheral benzodiazepine receptor regulates vascular endothelial activations via suppression of the voltage-dependent anion channel-1. *FEBS letters* **586**, 1349-1355 (2012).

4. Choi, S. *et al.* D,L-Sulforaphane-induced cell death in human prostate cancer cells is regulated by inhibitor of apoptosis family proteins and Apaf-1. *Carcinogenesis* **28** (1), 151-162 (2007).


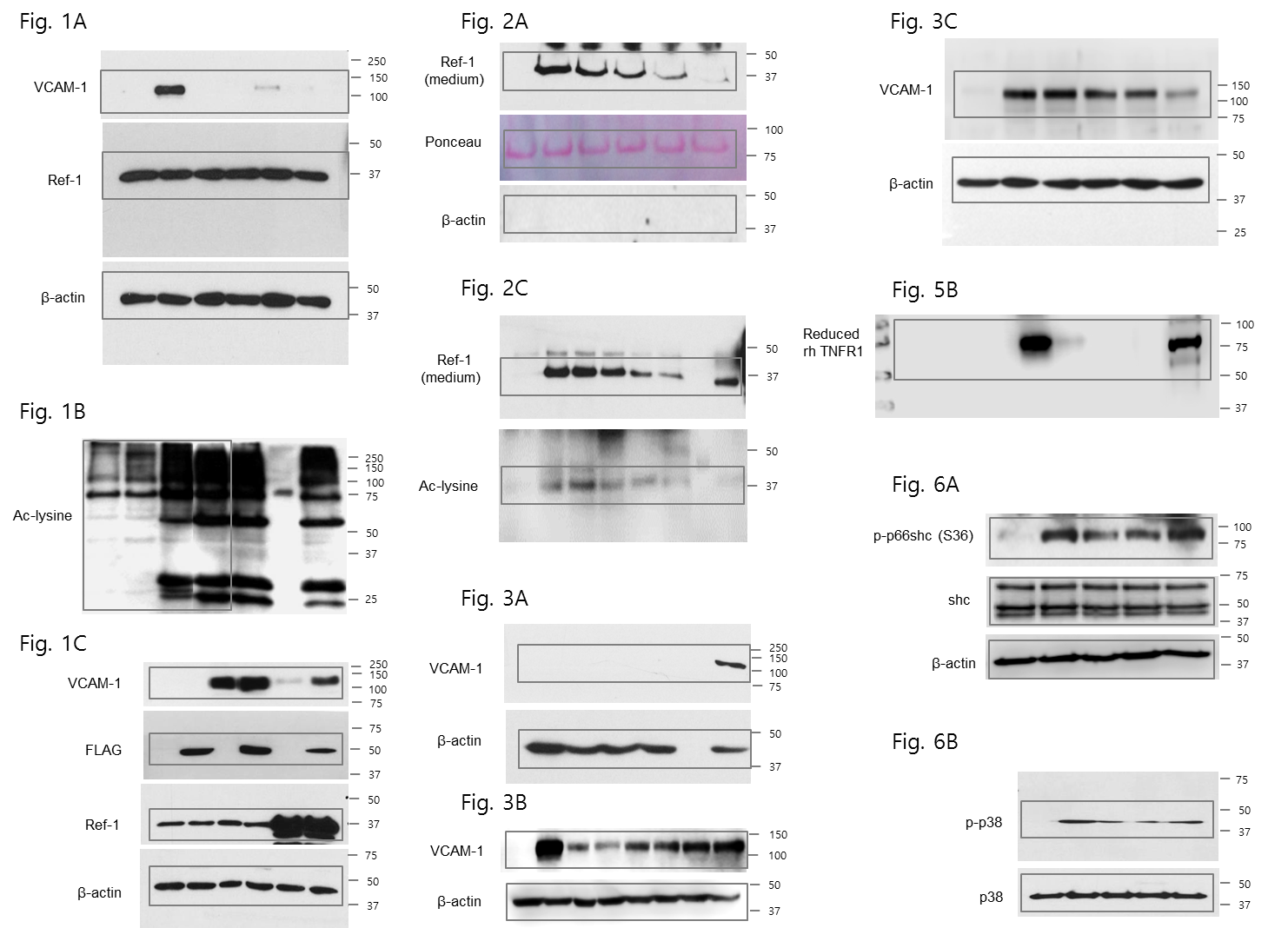


**Supplementary Figure 6.** Full length blots of the cropped VCAM-1, Ref-1, β-actin, Ac-lysine, FLAG(for Flag-tagged HDAC3), reduced rh TNFR1, phospho-p66shc(S36), shc, phopho-p38, p38 presented in the main Figures.


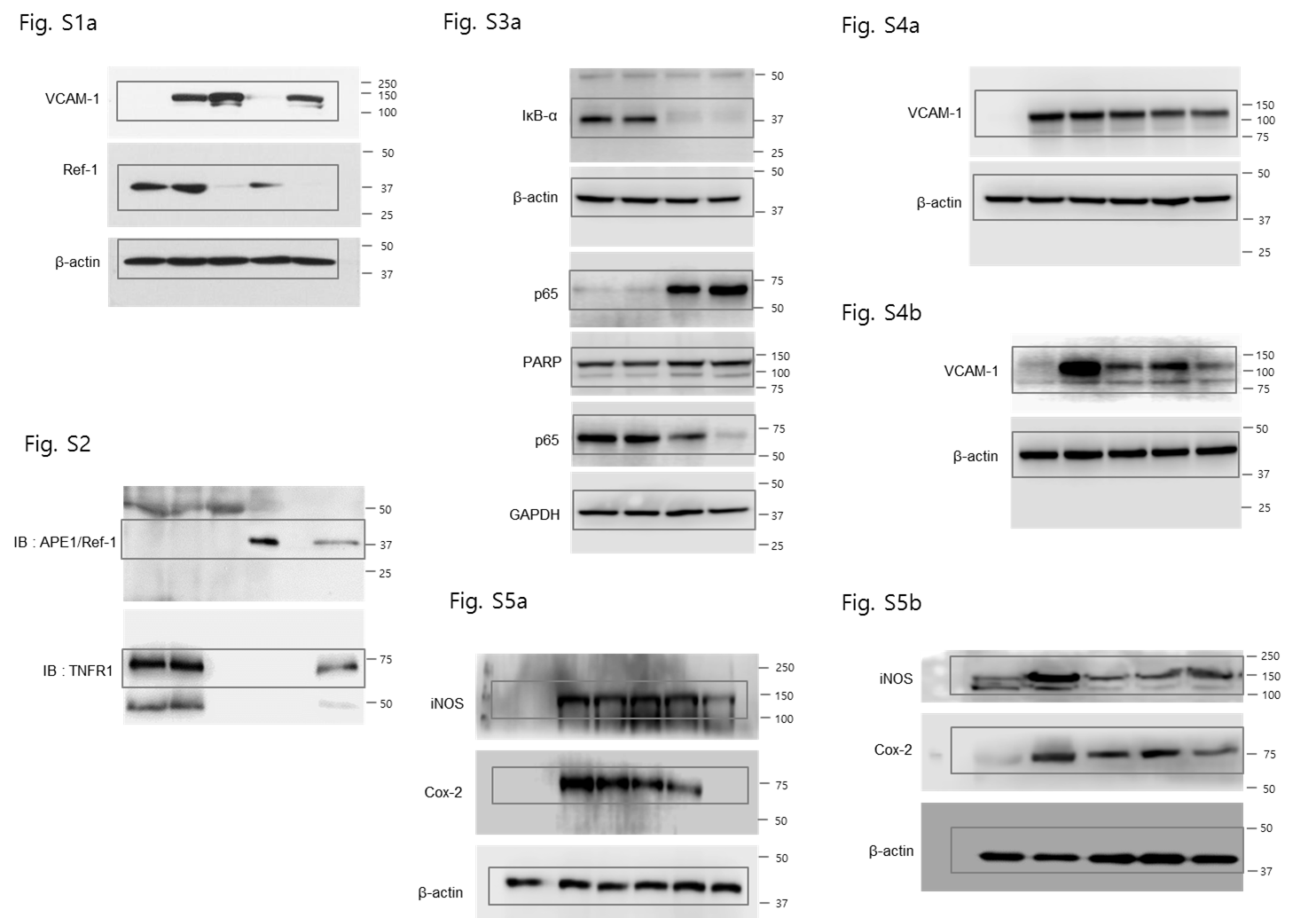


**Supplementary Figure 7.** Full length bots of the cropped VCAM-1, Ref-1, β-actin, TNFR1, IκB-α, p65, PARP, GAPDH, iNOS, Cox-2 presented in Supplementary Figures.
